# An Intelligent Framework for Cyber–Physical Satellite System and IoT-Aided Aerial Vehicle Security Threat Detection

**DOI:** 10.3390/s23167154

**Published:** 2023-08-14

**Authors:** Nazik Alturki, Turki Aljrees, Muhammad Umer, Abid Ishaq, Shtwai Alsubai, Oumaima Saidani, Sirojiddin Djuraev, Imran Ashraf

**Affiliations:** 1 Department of Information Systems, College of Computer and Information Sciences, Princess Nourah bint Abdulrahman University, P.O. Box 84428, Riyadh 11671, Saudi Arabia; namalturki@pnu.edu.sa (N.A.); ocsaidani@pnu.edu.sa (O.S.); 2 College of Computer Science and Engineering, University of Hafr Al-Batin, Hafar Al-Batin 39524, Saudi Arabia; tajrees@uhb.edu.sa; 3 Department of Computer Science & Information Technology, The Islamia University of Bahawalpur, Bahawalpur 63100, Pakistan; airgcu@gmail.com; 4 Department of Computer Science, College of Computer Engineering and Sciences, Prince Sattam bin Abdulaziz University, P.O. Box 151, Al-Kharj 11942, Saudi Arabia; sa.alsubai@psau.edu.sa; 5 Department of Software Engineering, New Uzbekistan University, Tashkent 100007, Uzbekistan; s.djuraev@newuzbekistanuniversity.uz; 6 Department of Information and Communication Engineering, Yeungnam University, Gyeongsan 38541, Republic of Korea

**Keywords:** aerial vehicles, autonomous vehicles, cyber-security, Internet of Things, machine learning

## Abstract

The small-drone technology domain is the outcome of a breakthrough in technological advancement for drones. The Internet of Things (IoT) is used by drones to provide inter-location services for navigation. But, due to issues related to their architecture and design, drones are not immune to threats related to security and privacy. Establishing a secure and reliable network is essential to obtaining optimal performance from drones. While small drones offer promising avenues for growth in civil and defense industries, they are prone to attacks on safety, security, and privacy. The current architecture of small drones necessitates modifications to their data transformation and privacy mechanisms to align with domain requirements. This research paper investigates the latest trends in safety, security, and privacy related to drones, and the Internet of Drones (IoD), highlighting the importance of secure drone networks that are impervious to interceptions and intrusions. To mitigate cyber-security threats, the proposed framework incorporates intelligent machine learning models into the design and structure of IoT-aided drones, rendering adaptable and secure technology. Furthermore, in this work, a new dataset is constructed, a merged dataset comprising a drone dataset and two benchmark datasets. The proposed strategy outperforms the previous algorithms and achieves 99.89% accuracy on the drone dataset and 91.64% on the merged dataset. Overall, this intelligent framework gives a potential approach to improving the security and resilience of cyber–physical satellite systems, and IoT-aided aerial vehicle systems, addressing the rising security challenges in an interconnected world.

## 1. Introduction

Over the past 20 years, pervasive environments have gained significant popularity, primarily due to the significance of pervasiveness and the intelligence of objects found in various environments, including buildings, towns, playgrounds, shopping malls, and more. Pervasiveness in an environment enables improved task control, efficacy, and efficiency by linking multiple devices and sensors. Additionally, pervasive environments allow for better event response and facility provision. Drone technology has advanced in recent years, resulting in the development of small-sized drones like quadcopters and mini drones [1,2,3]. These drones offer several advantages, including their ability to enter and hover inside buildings for monitoring and surveillance purposes. This feature is particularly useful for various fields, such as industrial area surveillance, military operations [4], catastrophe management, rescue missions [5], shipping services [6], precision crop sciences [7,8], and other miscellaneous applications. Commercial drones also have potential applications in areas like weather forecasting, and aerial photo and video shooting.

UAVs (unmanned aerial vehicles), commonly known as drones, are airborne machines that operate without human operators. These vehicles are frequently employed using aerodynamic forces to remotely pilot a machine [9]. Additionally, drones have impacted numerous industries and thus everyday life aided by their commercial usage. Drones collect and transmit aerial views and related data to base stations for informed decision making for monitoring and surveillance purposes [10]. However, the usage of drones on a mass scale in routine life has impacted lives in a variety of ways. It has hampered the safety and security of the masses and created a need for the regularization of policies regarding liability and the privacy of individuals as well as the public [11]. Small-sized drones are becoming increasingly prevalent in agriculture, shipping, and manufacturing due to their many advantages. The privacy and security threats posed by the widespread use of drones are major challenges and need to be addressed [12]. To make drones smarter, researchers have been exploring the addition of sensors that are small enough to be carried by these devices. Incorporating transmitters, sensors, and cameras can enhance the capabilities of drones, making them more useful and effective in a wide range of complex applications.

The defense and civil industries are the most benefited horizons of drone application. Inappropriate design and architecture make it vulnerable to security and privacy threats. New possibilities are created by the Internet of Things (IoT) and IoD despite posing security- and privacy-related challenges. Basic architecture and design changes are needed to address the privacy and security concerns posed by drone gadgets. Previously [1], layered architecture has been used to produce drones comprising a drone layer, an edge processing layer, a device connection layer, a data processing layer, a data storage layer, and a data visualization layer.

In an industrial drone design with multiple layers [1], the drone layer (DL) is the very first layer, via which a mini camera-equipped drone is connected to an IoT hub via an IoT gateway. This gateway plays a vital role in facilitating communication with base stations aided by a cloud-based IoT hub. The footage obtained from the drone is then transmitted to the data processing layer for analysis. The data storage layer is used to store the data analysis outcomes in the data centers for future reference and can be visualized by the data visualization layer. This architecture can be implemented using hub services and Microsoft’s Azure cloud storage. Data privacy and lack of support are the major drawbacks of this system in cyber-security. The IoDT (Internet of Drone Things) is a modern concept to integrate drones and IoT networks to enable connectivity between drones and IoT devices. This research introduces the concept of the IoDT [13] and highlights the connected security and privacy concerns.

Drones, like other IoT devices, are prone to security lapses and unauthorized access. Malicious actors may take advantage of a drone system’s weaknesses to compromise data security and privacy. In addition, concerns regarding data security and possible misuse are raised due to the transmission and gathering of enormous volumes of data by drones. A robust security system is required to unlock the full potential of the IoDT. The proposed research is largely concerned with increasing the cyber-security of drones and IoT devices. To ensure the security and privacy of this smart drone gadget, this research proposes the implementation of blockchain technology. This framework is based on seven layers, including an edge processing (EP) layer, a drone layer (DL), a data storage layer, a data connection layer, a security and privacy layer, a data processing layer, and a data visualization layer. In addition to standard drone operations, this layered approach includes data security and analytic techniques. Machine learning (ML) models are employed to improve the security of drones. The primary objectives of the proposed architecture in the current study are as follows:

- The study discusses the most recent developments in drone safety, security, and privacy, as well as the Internet of Drones (IoD), highlighting the need for secure drone networks that are resistant against hacking and other intrusions.
- The proposed framework incorporates advanced machine learning models into the design and structure of IoT-aided drones to prevent cyber-security vulnerabilities. This integration improves the flexibility and security of the technology.
- This research work involves the construction of a new dataset performed by merging a drone dataset and two benchmark datasets (KDD CUP 99 and NSL-KDD). This new dataset serves as a valuable resource for further analysis and evaluation of drone-related algorithms and techniques.
- The proposed strategy surpasses previous algorithms by achieving 99.89% accuracy on the drone dataset and 91.64% on the merged dataset. This demonstrates the effectiveness of the intelligent framework in enhancing security and resilience in cyber–physical satellite systems and IoT-aided aerial vehicle systems.
- The intelligent framework presented in this research article offers a means to improve the security and resilience of various systems, including cyber–physical systems, satellite systems, and IoT-aided aerial vehicle systems.

Section 2 of this research paper discusses the existing studies related to identifying vulnerabilities and loopholes in targeting IoT systems and drones. A limited number of studies found that the incorporation of authentication methods can enhance the security of these drones. In Section 3, the drone architecture and the layered framework for foolproof drone systems are described. Section 4 covers the topics of authentication and access control related to drones. The trials and their upshots are presented in Section 5, while Section 6 presents the conclusions and recommendations for future endeavors.

## 2. Related Work

Drones are commonly utilized for military and defense applications. They come in a range of sizes, from large, 200-foot war machines to small, inch-wide micro drones that fly through the air. Size is a critical factor in determining the appropriate use of a drone. Additionally, the flying range of a drone can vary significantly depending on the type, with some advanced military drones being capable of flying up to 17,000 miles without the need for ground control. Maximum flight time also varies based on factors such as altitude, surface area, and terrain. Drones can fly at varying heights, from just a few meters off the ground to as high as 65,000 feet [1].

### 2.1. Threats to Drone Security

Drone security measures comprise multiple layers and types, which are dependent on their usage, size, and control techniques. Typically, drones utilize an IEEE 802.11-based [13] Wi-Fi communication protocol [14] and include both ground stations and a Wi-Fi network. Due to the absence of encryption technologies in the drone, these gadgets are vulnerable to cyber-attacks and hijack [4]. Man-in-the-middle attacks, which can occur within a range of 2 km, are also a common method used to hijack drones [15]. In the military industry, the IoD has become increasingly popular, posing a challenge to privacy and security concerns during design [16]. To ensure data protection, privacy issues such as data accessibility, information leakage, encryption, and decryption techniques need to be addressed [17]. In recent years, researchers have identified four categories of security threats related to sensor- and protocol-based threats, jammers, and conceded components. Table 1 presents a literature review of these categories.

Table 1 demonstrates the review of the literature that primarily focused on identifying cyber-security loopholes in drones, with limited discussions on potential solutions. One potential research avenue has been to utilize encryption algorithms to ensure safe and secure data transmission between the drone and its base station [16]. Small drones have obtained popularity due to their size and likely peril to the government and general public’s data privacy [26]. Researchers also established a risk challenge for drones [14,27,28,29,30]. For instance, Tian proposed an operative and smart validation model for the IoD assisted by edge, ensuring the drone networks’ data-related security [31]. Similarly, a system was presented by Hell to ensure the safety of drone data in a commercial industrial/factory area [2]. In 2019, a gas leakage-sensing drone idea was projected by the authors to ensure timely action to curb the fatal scenario [3]. Drones are mainly used for monitoring in the agriculture and security fields.

Over the last decade, drone-related security threats are the talk of the town in the research arena. The privacy issues associated with smart-city drone applications are discussed in [19], and Table 1 highlights other important issues. Drone network attacks, prospects, and limitations are also the interest areas of researchers in the cyber-security domain [32]. The business sector has similar challenges and applications, as presented by similar studies [5,33,34] using blockchain/crypto technology using 5G and drones based on the IoT for the safe transmission of data [34]. This system has limitations in manually identifying the intensity and nature of the threats. A secure and smartly effective drone system with the ability to investigate attacks and implement security measures for drone data integrity is the need of the hour. Some studies have attempted to solve device authentication problems by using key agreement [35] and key-enabling data [6] for secure drone data delivery. Commercial drones [6,35,36,37] are facing the general issue of the hijacking of drones, UAVs, and drones in the agricultural sector [22,38] aided by the IoT. Solutions to these general issues are proposed in [7] and [8]. Another concern relating drones and UAVs is GPS (global positioning system) tracking [39], which requires robust, authentic, and foolproof resolution. Drone interception and hijacking are also part of the studies carried out in this domain [23,24,25,40].

### 2.2. Implementation of Drone Security with Machine Learning

ML techniques are classified into semi-supervised, supervised, and unsupervised categories. Cloud computing [41], mobile networks [42], IoT systems [43], and sensor-based wireless networks [44] are the areas where ML models have been widely used by researchers to handle cyber-attacks. For example, self-learning models were combined with supervised learning models by Vedula et al. [45] to use two features to detect DDoS attacks. They combined LSTM Autoencoder and RF classifier. For the scenarios of all and sparse traffic, their window identification approach achieved accuracy rates of 94% and 93.5%, respectively.

ML techniques are classified into semi-supervised, supervised, and unsupervised categories. Cloud computing [41], mobile networks [42], IoT systems [43], and sensor-based wireless networks [44] are the areas where ML models have been widely used by researchers to handle cyber-attacks. For example, self-learning models were combined with supervised learning models by Vedula et al. [45] to use two features to detect DDoS attacks. They combined LSTM Autoencoder and RF classifier. For the scenarios of all and sparse traffic, their window identification approach achieved accuracy rates of 94% and 93.5%, respectively. Their proposed hybrid LSTM-RF model showed the best results, with a window size of 100.

No research on ML model usage in drone networks for cyber-attack recognition was found. However, another study suggested a probabilistic approach in a constrained cyber–physical system to control and detect actuation attacks [46]. Their research was primarily concerned with the PA2 attack, in which the attacker blocks communication between the actuators and the controller. Based on a hypothesis-testing methodology, a group of parallel detectors was suggested. The detection and control goals were written as two distinct stochastic objective functions using a probabilistic technique to cope with uncertainty. The authors also proposed a drone security access control system and have previously used ML for wireless networks (wi-net) security systems, as shown in Table 2.

The literature review suggests that there is a need for a comprehensive solution to address cyber-security threats and ensure the safety of drone data. While many studies have identified the challenges and issues related to drone security, few have proposed effective solutions to mitigate these risks [34]. The use of machine learning models has shown promise in dealing with cyber-attacks in various networks, but there is a lack of research on their application in drone networks. Additionally, the authentication system proposed in some studies may not be suitable for IoT-based drone networks. Therefore, this research gap needs to be addressed to make drones compliant for the industry and for commercial use while ensuring their security and privacy.

Existing studies related to drone security have certain limitations that need to be addressed. First, the architecture and design of small drones have not received sufficient attention, resulting in vulnerabilities that can be exploited by potential attackers. Additionally, the current data transformation and privacy mechanisms of small drones may not align with the specific requirements of the domain, leaving them susceptible to security breaches. Furthermore, while the Internet of Things (IoT) is utilized for inter-location services in drones, there is a lack of comprehensive research on establishing secure and reliable networks for optimal drone performance. Moreover, previous studies have not fully explored the integration of intelligent machine learning models into the design and structure of IoT-aided drones, which could enhance their adaptability and security. Overall, these limitations highlight the need for further research and development to overcome security challenges and ensure the resilience of drone systems in an interconnected world.

To ensure drone security, a smart vigilant system is a prerequisite to investigate the attacked data automatically and take corrective measures according to the scenario and the situation at hand without in-person interference. ML models have previously been deployed for mobile-based and wireless sensor-based networks for cyber-security, but they are yet to be applied to the security of drone-based vehicles. This study addresses the issue of access control authentication methods for drones with an ML-based solution.

## 3. Drone Architecture

**Figure 1:**
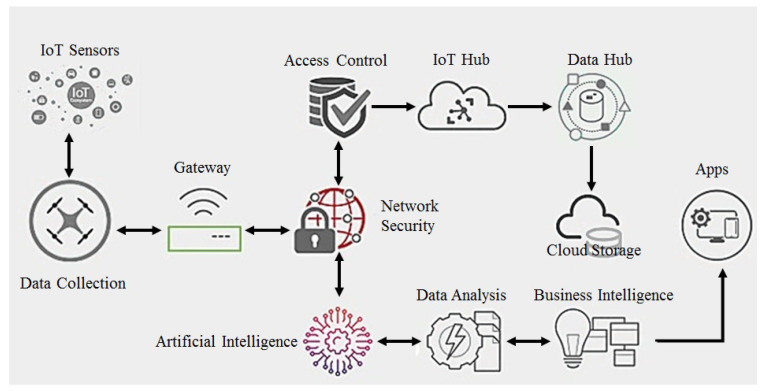
Smart security drone-layer-wise architecture.

The main focus of this research is to enhance the cyber-security of IoT drone devices, particularly small drones, by improving their basic framework. Privacy threats, cyber-security concerns and interception chaos prevention, and reliable security are the aims of this study. A layered approach is a framework that addresses analysis methods and security issues in each layer, ensuring added data security in conventional drone operations. This layered architecture enables the easy upgradation of the anticipated method. Machine intelligence through ML models is incorporated to enhance drone data security. Figure 1 illustrates the proposed framework.

The emergence of tiny drones has initiated the latest promises in civil and defense activities. The lack of advanced architecture and design has made these modern gadgets susceptible to security and privacy threats. Advancements in the IoD and IoT have presented innovative prospects but with additional security and privacy challenges. The current context is inadequate in terms of ensuring data privacy and security, thus making it unreliable.

### 3.1. Layered Architecture for Secure Smart Drones

The architecture commonly used for smart drones [1], illustrated in Figure 1, is a layered design. To enhance its security and privacy features, a new layer is added, while the layer processing data is updated with components related to machine intelligence.

The drone data layer is the first layer in an industrial drone’s architecture having a camera-equipped mini drone or quadcopter. IoT sensor data update this layer. Sensors like cameras, GPS, and radar types of advanced sensors are employed. It enables a drone to sense and capture data and communicate them to the subsequent layer.

The next layer comprises a UAS (unmanned aircraft system) drone responsible for data capturing and flight operations of drones. A UAS drone includes a communication connection and a ground controller. Phantom 3 drones by DJI Company (Shenzhen, China) are based on these suggested design and architecture with a communication link and a customized remote controller to control the flight, and auxiliary sensors can also be attached as needed in this architecture.

In the edge processing drone layer (EPL), IoT-based unprocessed raw data and drones are sent to the PL (privacy layer). Here, data authentication is ensured. The layer manages data transmission and communication with the subsequent layer, which is the cloud layer. Various gateway devices that facilitate wireless communication exist, with Wi-Fi being a fast transmission option. This layer efficiently handles device-to-cloud communication and flooding, data protection, and cashing. Cloud communication is performed through the Azure IoT gateway as recommended by research, with the IoT gateway’s design.

The privacy and security drone layer ensures the authentication of the device and secures access using ML prototypes. It implements data safety and security, which are crucial elements of the IoT framework. At this level, privacy threats are identified and addressed, as they pose a significant risk to the system. The following are privacy threats that can occur in this stage:

- Physical privacy threat, which refers to the unauthorized access or tampering of physical devices, sensors, or drones.
- Behavioral privacy threat, which pertains to the collection of personal data through user behavior tracking or monitoring.
- Location privacy threat, which concerns the tracking or disclosure of an individual’s location without consent.

To address these security risks, various authentication schemes and protocols must be implemented. Unauthorized individuals may use several security breaches to cause such threats, including intrusion, spoofing, jamming, and DoS attacks. In the proposed architecture, a machine learning algorithm is utilized to maintain device authentication and detect potential security attacks. This allows users to be alerted and take action to prevent such threats from occurring.

In the drone device connection layer, IoT gateways are the backbone of such systems in linking base stations to a cloud-based hub in the IoT domain. Added security orchestration and automation are ensured for connectivity to the authenticated devices only by using an additional module. This hub ensures the inter-communication between IoT devices and applications as a message medium enabling bidirectional communication between cloud systems and IoT network-based IoT devices. In this layer, security arrangements are made to allow only authenticated devices to access the network. Sensor data from tagged networks and drones are sent to the crypto/blockchain technology client, which ensures data reliability and storage in a cloud server database. A simple blockchain procedure is used to ensure real-time security for devices and IoT networks.

Once acknowledged at the IoT hub, data are transmitted to the drone data processing layer for analysis. A machine intelligence module and a data hub service are the two new modules implemented in this layer. There are several available ML algorithms that can be selected based on the situation and data requirements. The intelligent ML approach to device authentication is the focus of this research. In this layer, the Naive Bayes model, which is an intelligent ML algorithm, is used as an authentication method. The IoT hub layer, in this stage, authenticates devices by timestamping drones’ data at stipulated intervals of time. These data from drone flights are used for testing and training the model. The model undergoes training before undergoing testing to determine its capability to detect malicious drone activities. In the case of mixed drone data, the system receives an alert and inhibits communication with the cloud server. Inappropriate drone behavior triggers the machine intelligence module to detect it and disallow unauthorized access. Numerous security risks are linked to flight operations, with interception attack, where an outsider takes control of the drone, being the most prevalent. Another risk is wrong information from unauthorized access to drones being spread by unauthorized individuals.

This architecture employs the Naive Bayes classifier to train a model, which is then utilized to validate newly generated aircraft trajectories. To assess accuracy, precision, and recall, the dataset named KDD’99 and an instantaneous dataset were utilized. Precision refers to the percentage of genuinely correct and accurate predictions, while recall refers to the percentage of incorrect and accurate predictions.

The data analysis results obtained by the drone data storage layer are archived and stored in data centers in the DSL. NoSQL, a cloud database, is used to store drone-generated results in the DL (drone layer). The data include drone information, data from drone sensors, and networks. NoSQL databases provide schema-less data storage, enabling quick access and retrieval of information. This technique can handle storing a large number of data. NoSQL databases are self-indexing, making them more practical and preferred over SQL databases. Widely used database storage structures are graph, key-value, document, and column structures.

The drone data analysis layer provides numerous tools and services for data monitoring. The platform utilizes Azure services by Microsoft. DVL results are viewed on a mobile app presenting the intelligent model’s predictions about the security level (SL) of a drone. The Naive Bayes model intelligently identifies drone attacks. The BI architecture, in which stream analytics outcomes are used by the MS Power BI app, is stored in a data center for real-time modeling and data visualization employing business intelligence upshots.

### 3.2. Physical Components

Readily available and inexpensive peripherals were utilized in the trials. A Mega 2560 microcontroller by Arduino (Somerville, NJ, USA) with an integrated Wi-Fi module, ESP8266, was used as the processing device to sense data.

Numerous size and shape options are available when it comes to drones, and these options are deployed on a use case basis. Phantom 3 Standard, a robust vehicle manufactured and marketed by DJI, was used in this experiment. A wirelessly connected custom controller is used to operate it in remote locations. Tracking, locating, and identifying objects are performed by using radar sensors in remote locations. Electromagnetic energy transmission is used by sensors to operate toward target areas and objects. The detection accuracy of radar sensors is superior to that of optical sensors. Alternatively, radar sensors can also be replaced with accelerometers. In our recommended system, we used an ultrasound-based proximity sensor, HC-SR04, for this purpose. Patterns for objects are calculated by this sensor.

GY-GPS6MV2 (UBLOX, Zurcherstrasse, Switzerland) is a device for receiving GPS signals with a NEO-6M chip embedded onboard. A battery-connected LED light turns on while transmitting or receiving GPS data. This module is sensitive up to −161 dBm.

The BMP180 sensor is a low-battery-consuming module providing pressure and altitude measurements for a particular location. It has high accuracy in a very compact size. This is an OEM module, more precise than other altitude and pressure measurement sensors.

The widespread use of ZigBee wireless transmission technology is due to its unique features, including its ability to transmit both analog and digital data. In this context, the XBee Pro S1 module was utilized for its long-distance data transmission capabilities.

## 4. Drone Security

To ensure the security of drones, an efficient system is needed to analyze attack data and take proactive measures to maintain the security of drones. For the development of a reliable and trustworthy system, security, reliability, and consistency are critical factors in the Internet of Drones (IoD). Although ML models have previously been used for cyber-security in sensor-based wireless networks and mobile-based networks, they have yet to be thoroughly applied in drone-based security. Therefore, this study suggests an ML-based solution for authentication and control access methods for drone security.

Cyber-security systems can be evaluated using various metrics that are effective in handling diverse performance indices. In this study, we suggest utilizing the following performance evaluation parameters for the projected system [58]:

- Threat exposure for cyber-security.
- DDos (denial of service) attacks.
- Malicious attacks.
- Jamming.
- Spoofing.

These cyber-security metrics are utilized to enhance the evaluation process of the system’s effectiveness. This study contributes significantly to access control and foolproof authentication for IoT devices and drones by introducing an ML-based research solution. This research primarily addresses the research gap and enhances the safety and reliability of drones against significant cyber-security issues, converting them into commercial and industrial monitoring tools. As described in Section 3, this is a seven-layered drone security system. Data collected from the DL and EPL undergo protocols related to security and privacy in the SPL (security and privacy layer) before transferring to the device connection (DC) layer. The use of machine learning models in the security and privacy layer ensures data protection from potential identity and privacy threats. In the event of an attack being detected, a mobile alert is sent.

### 4.1. Communication Security Threats

Unmanned aerial vehicles (UAVs) offer numerous advantages as technology progresses, but they are also subject to limitations and concerns regarding privacy, security, and safety [59,60]. Implementing regulations and licensing measures for drone usage is crucial to restricting unnecessary aerial photography. Authorities worldwide enforce strict policies to combat uninformed aerial photography. In terms of network security and risk analysis, the coverage of UAVs differs significantly from wireless sensor networks (WSNs) or mobile ad hoc networks (MANETs) due to resource constraints and wider coverage [61].

The framework governing drone operations in a given vicinity is referred to as authentication, authorization, and accounting (AAA). It grants certain privileges to drone controllers based on administrative rights and imposes stringent authentication procedures to prevent diversion to unknown entities. It also facilitates tracking down drone operators in case of uncertainty or illegal activity, thereby limiting illegal surveillance, cyber-attacks, and privacy threats. Various mechatronic engineering solutions have been proposed to address these malicious activities [62].

The availability of low-cost drones in the market raises concerns about their potential misuse for criminal activities. Their capacity to carry external payloads increases their danger, as they can transport hazardous chemicals or explosives unnoticed [63]. Furthermore, their ability to access hard-to-reach areas poses a significant risk. Safety concerns arise when drones fly over populated areas due to the potential for accidents or crashes leading to tragic incidents [64]. Notable incidents, such as the collision between a UAV and a passenger aircraft (British Airways BA727) in April 2016, highlight these risks. In light of these incidents and issues, several public safety measures are recommended:

- Incorporating a reset option to allow drones to hover in case of hacking or deviation from their designated path due to strong winds, enabling regaining control.
- Developing drone filters capable of detecting signal jammers that could potentially control the drones for cyber-attacks.
- Addressing privacy concerns associated with high-definition cameras on UAVs, ensuring that the recording of private property without permission is prohibited. Canadian Public Safety (CPS) has explicitly stated the need for mutually agreed-upon permission before drones can fly over private properties [65].

### 4.2. Proposed Approach

This section presents a comprehensive approach to augmenting the security system of drones, encompassing both hardware and software components. The design of the proposed framework integrates cutting-edge technologies to address security and privacy concerns in drone operations. Additionally, the datasets utilized in the experiments and the machine learning models employed in this research are thoroughly described to ensure transparency and reproducibility.

**Figure 2:**
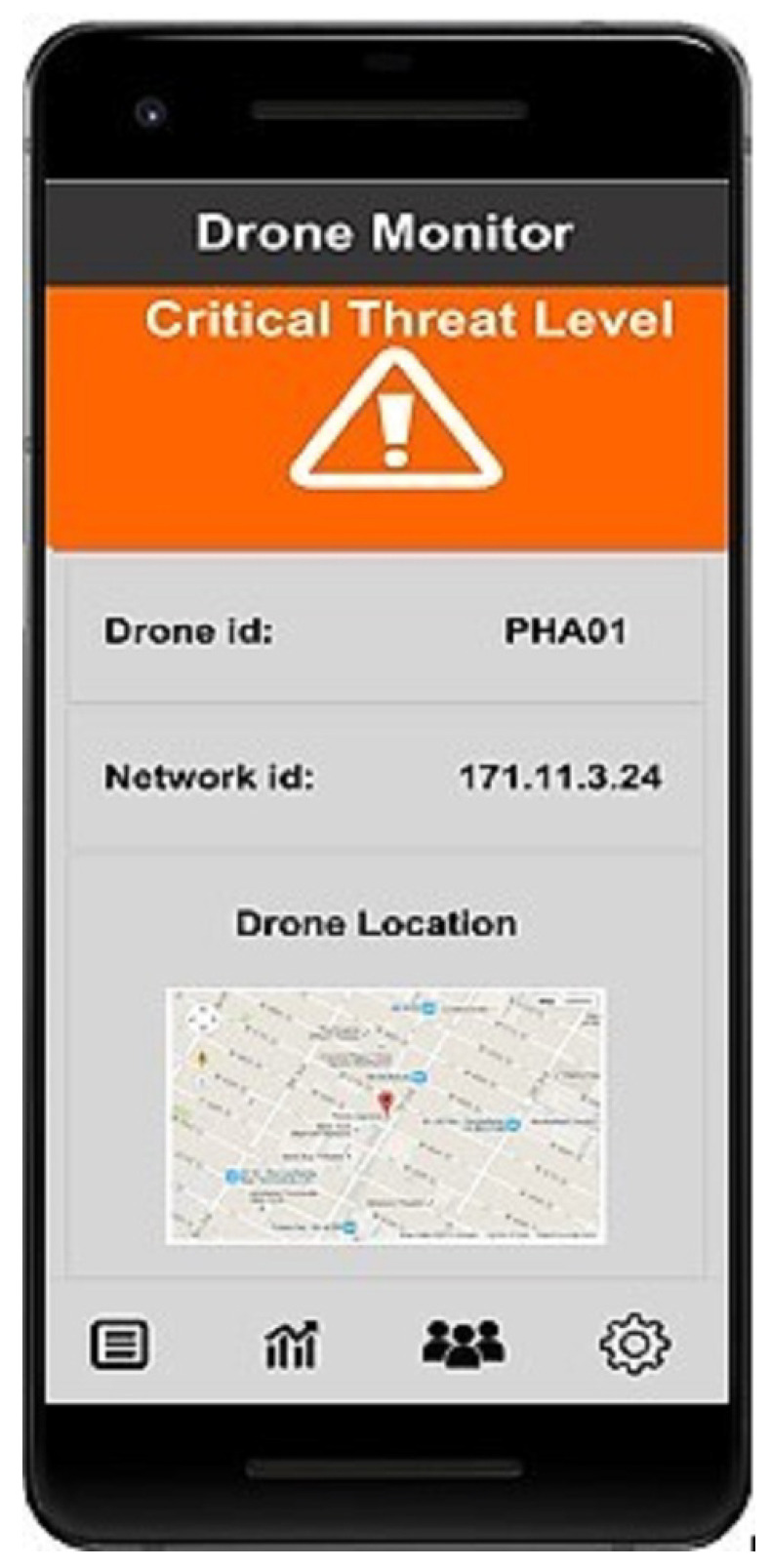
Mobile identification of an attack.

The drone security system’s architecture comprises seven layers. Information flows from the drone layer and edge processing layer to the security and privacy layer, which safeguards the data against security threats using machine learning models. Internal authentication is provided using the edge processing layer; afterwards, the data are transferred to cloud storage, where access control is provided using the Microsoft Azure authentication protocol. In the Microsoft Azure cloud storage, a trained model is already placed using the drone data integrated with NSL-KDD, STIN, and KDD CUP data. This model makes predictions about attacks, and when an attack is detected, a mobile alert is triggered. Figure 2 illustrates an example of a mobile alert indicating the identification of an attack within the system.

#### 4.2.1. Smart Drone Components

The smart secure drone is built using the following components: Mega 2560 microcontroller, **HC-SR04 ultrasonic sensor, GY-GPS6MV2 GPS module, BMP180 barometric pressure sensor**, and XBee Pro S1 wireless communication module. The components are sourced from (Amazon, 410 Terry Ave N, Seattle, WA, USA). Initially, the Mega 2560 microcontroller serves as the brain of the drone, providing the necessary processing power and control capabilities. It communicates with other components, collects sensor data, and executes flight control algorithms. The Mega 2560 microcontroller’s current time is noted for collecting drone runtime data, flight duration, and port attack URL_ID. The HC-SR04 ultrasonic sensor is used for obstacle detection and avoidance. It emits ultrasonic waves and measures the time it takes for the waves to bounce back, enabling the drone to detect nearby objects and adjust its flight path accordingly. It takes decisions like relocation and positioning changing based on that. The GY-GPS6MV2 GPS module provides accurate positioning and navigation data. It receives signals from GPS satellites to determine the drone’s latitude, longitude, altitude, position ID, and accurate x and y angle values. This information is vital in flight planning, waypoint navigation, and tracking. The BMP180 barometric pressure sensor measures atmospheric pressure, allowing the drone to estimate its altitude with high precision. These data are essential to maintaining stable flight and performing altitude-related tasks. This sensor also determines the pressure exerted on the drone. The XBee Pro S1 wireless communication module facilitates secure communication between the drone and a ground station or other devices. It enables real-time data transmission, remote control, and the monitoring of the drone’s status. By integrating these components, the smart secure drone combines advanced sensing capabilities, reliable navigation, and secure communication to ensure safe and efficient operation.

#### 4.2.2. Dataset

Real-time data from drones that included GPS-based features such as longitude, latitude, and altitude, along with drone OBD data and KDD intrusion detection features (data are available at: https://github.com/MUmerSabir/MDPIElectronics accessed on 19 May 2023) were used in this experiment. This prototype was trained and tested to verify the intended results using the drone dataset and a couple of related benchmarks for invasion uncovering and cyber-security attack forecast. Table 3 displays the dataset classes.

#### 4.2.3. Learning Models

ML plays a significant role in enhancing the accuracy of prediction rating based on reviews. Various ML classifiers are available for ranking classification, and the Python Scikit-learn library offers a plethora of rich variants. An open-source platform having a great user support base is a characteristic of Python Scikit-learn. All the classifiers used in this study were implemented using the Scikit-Learn library. The models used as baseline are presented in Table 4.

The hyperparameter values employed for all machine learning models used in this study are presented in Table 5.

The proposed approach combines two models, an ML model and a simple neural network model. The LR algorithm [71] is a statistical approach that examines the data and variables used to predict results. It is an effective method for classification tasks with low variance. This model can also extract features from the data. Updating the model with new data is easy by employing Stochastic Gradient Descent.

A simple deep learning prototype, Multilayer Perceptron (MLP), demonstrates reasonable classification performance. It consists of multiple layers, where features are indicated by input layer neurons, while hidden layers process input data using weights to feed into the output layer, where the output value is represented by the neurons. Optimal results are obtained by selecting the neurons and hidden layers as per requirements. To develop classification training efficiency, appropriate hyperparameters are used to train the model. Gradient Descent-based backpropagation is generally used to manage MLP layer weights. Rectified Linear Unit (ReLU) is commonly used as the activation function in the hidden layers, while sigmoid is used as the activation function (f(x)) in the final layer.

(1) $$f\left(x\right)=\frac{1}{\left(1+e^{\left(-x\right)}\right)}$$

Voting classifiers combine the results of various classifiers to make a final decision based on voting. There are two types of voting classifiers: soft- and hard-voting classifiers. The weight percentage of each classifier is computed using soft voting, while classifiers’ result prediction is performed using hard voting. For every entry, class probability multiplied by classifier weight and then averaged to determine the final result is predicted by this prototype. In our research, a voting classifier, Logistic Regression, and Multilayer Perceptron (MLP) are used in combination, outperforming other tactics applied individually for intrusion detection. Algorithm 1 illustrates the methodology of the projected voting classifier, presented as follows:

(2) $$\hat{p}=argmax\left\{\sum_{i}^{n}LR_{i},\sum_{i}^{n}MLP_{i}\right\}.$$

where $\sum_{i}^{n}LR_{i}$ and $\sum_{i}^{n}MLP_{i}$ predict the probability-based results for each test model using Logistic Regression and Multilayer Perceptron, respectively.

**Figure 3:**
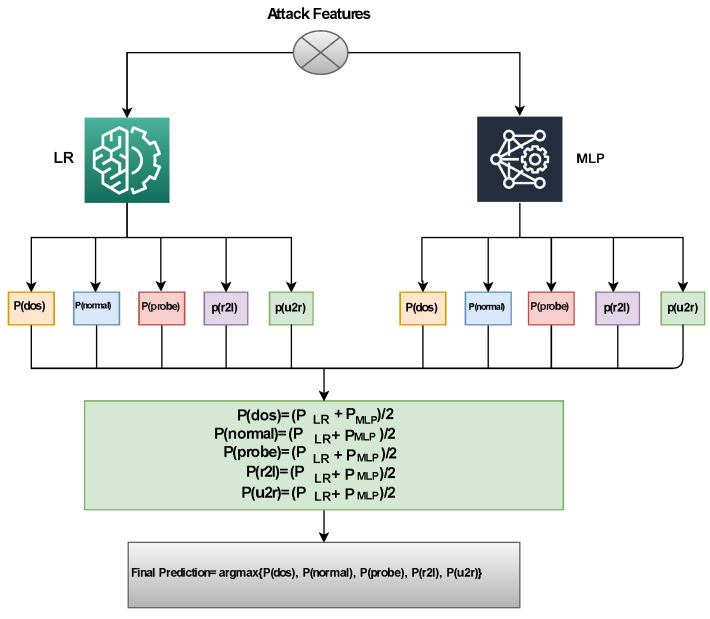
RegressionNet architecture.

Figure 3 shows the visual representation of the proposed ensemble model. Logistic Regression and Multilayer Perceptron instance probabilities are passed through soft voting criteria in Algorithm 1.
**Algorithm 1:** Ensemble approach using LR and MLP (LR-MLP).**Input:** input data ${\left(x,y\right)}_{i=1}^{N}$$M_{LR}$ = Trained_ LR$M_{MLP}$ = Trained_ MLP      1:**for** i=1toM**do**      2:   **if** $M_{LR}\neq 0\;\&\;M_{MLP}\neq 0\;\&\;training\_set\neq 0$ **then**      3:     $ProbLR-dos=M_{LR}.probibility\left(dos-class\right)$      4:     $ProbLR-normal=M_{LR}.probibility\left(normal-class\right)$      5:     $ProbLR-probe=M_{LR}.probibility\left(probe-class\right)$      6:     $ProbLR-r2l=M_{LR}.probibility\left(r2l-class\right)$      7:     $ProbLR-u2r=M_{LR}.probibility\left(u2r-class\right)$      8:     $ProbMLP-dos=M_{MLP}.probibility\left(dos-class\right)$      9:     $ProbMLP-normal=M_{MLP}.probibility\left(normal-class\right)$     10:     $ProbMLP-probe=M_{MLP}.probibility\left(probe-class\right)$     11:     $ProbMLP-r2l=M_{MLP}.probibility\left(r2l-class\right)$     12:     $ProbMLP-u2r=M_{MLP}.probibility\left(u2r-class\right)$     13:     Decision function = $max(\frac{1}{N_{classifier}}$ $\sum_{classifier}(Avg_{\left(ProbLR-dos,ProbMLP-dos\right)}$      
$,(Avg_{\left(ProbLR-normal,ProbMLP-normal\right)}$      
$,(Avg_{\left(ProbLR-probe,ProbMLP-probe\right)}$      
$,(Avg_{\left(ProbLR-r2l,ProbMLP-r2l\right)}$      
$,Avg_{\left(ProbLR-u2r,ProbMLP-u2r\right)}))$     14:   **end if**      15:   Return final label $\hat{p}$     16:**end for** 

## 5. Results and Discussion

This section explains the results obtained from the experimentation conducted after presenting a list of suggested algorithms and sensors in the previous section. The results showcase the security prominence of the drones and the IoT network identified using machine learning in the mobile system. Four measures were used to evaluate and compare the performance of the prototypes, with the confusion matrix serving as a tool for calculations. The confusion matrix includes True Positive (TP), False Positive (FP), True Negative (TN), and False Negative (FN) elements. Table 6 presents the performance measures used in this study.

### 5.1. Experimental Results

This section presents the results of the experiments conducted. The proposed model’s performance is compared and evaluated with other state-of-the-art ML prototypes employed on the drone-based dataset. The dataset was divided into training and testing sets in a ratio of 70:30. The experiments were conducted on a Dell PowerEdge T430 GPU, which has an 8 GB capacity graphics card, along with 2x Intel Xeon eight-core CPUs running at 2.8 GHz and 32 GB DDR4 RAM. The experiments took place within the Jupyter Notebook environment, utilizing the Python programming language and Anaconda. Further details are shown in Table 7. The researched classifiers included Random Forest, MLP, Logistic Regression, Decision Tree, Naive Bayes, and RegressionNet (which is a voting ensemble of Logistic Regression and Multilayer Perceptron). All activities were conducted using Python and implemented using Keras, Sklearn, and Tensorflow. The data used in the study were divided into three categories, namely, jamming, spoofing, and DOS attacks. The achieved accuracy score for this task was higher than 99%, the highest score obtained in accuracy in controlling processes related to cyber-security.

The drone dataset was comparatively analyzed with respect to the classifiers, and the results are presented in Table 8. The results reveal that both ML and simple deep learning prototypes demonstrated substantial success in intrusion detection on the drone dataset. Table 8 shows that NB displayed the lowest recall, precision, accuracy, and F1 score results. However, a slightly better accuracy was shown by MLP, at 99.64%. Furthermore, DT, RF, and SVM attained more than 99% value in terms of all evaluation measures. The suggested RegressionNet method demonstrated strong results, with 99.80% values in terms of F1-score, precision, accuracy, and recall, in classifying attacks into Prob, DoS, R2L, and U2R on the drone dataset.

The analysis of Table 8 reveals that the voting group of the two top-notch models could classify attacks effectively into four categories—U2R, R2L, Prob, and DoS—with 99.80% accuracy. Additionally, the graph compares the performance of the drone dataset with the system data. The drone data are utilized by the ML model to generate alerts and identify cyber-attacks.

### 5.2. Validation of the Proposed Approach

In this section, we investigate the significance of the proposed RegressionNet model using two further datasets. The first dataset is the benchmark STIN security dataset, and the second one is our own developed security dataset, which is a combination of features and attack types of the KDD CUP 99, NSL-KDD, and STIN datasets [72]. The purpose is to extend the feature set and develop a database in which training models get trained on all types of attacks at once. The STIN security dataset [73] includes nine terrestrial and a couple of satellite attacks. Flow-based features were utilized to construct this dataset. Table 9 outlines the dataset’s characteristics while Table 10 highlights the accuracy of the classifiers on STIN security and merger of all datasets.

### 5.3. Performance Comparison of Proposed Approach and State-of-the-Art Models

Table 11 provides an accurate comparison of the anticipated voting classifier with advanced models from the literature review. Various techniques have been implemented by researchers, such as PCA + MCA, SVM-ANN, and DT-RFE, to improve the models’ performance in intrusion detection, while the latest deep learning methods, such as the Deep Hierarchical Model, have also been utilized. Nevertheless, the suggested approach outperformed others with an accuracy score of 99.89% in intrusion detection.

To demonstrate the strength and generalizability of the offered methodology, experiments were conducted on the NSL-KDD [79] and KDD Cup 99 [80] datasets, as shown in Table 11. The model RegressionNet outperformed the rest of the models from the literature on both datasets, indicating its supremacy in intrusion detection.

### 5.4. Discussion

Unlike previous studies that might have primarily concentrated on single-layered architectures for drone systems, the proposed framework introduces a multi-layered approach that incorporates advanced machine learning models for enhanced security. The integration of machine learning not only boosts the robustness of the technology but also enables adaptive responses to evolving cyber-threats.

A new dataset is constructed by merging drone data with benchmark datasets, namely, KDD CUP 99 and NSL-KDD. This new dataset serves as a valuable resource for benchmarking and validating the efficiency of the proposed algorithms in a diverse range of attack scenarios.

The intelligent framework achieves high accuracy and offers the potential for improving security in interconnected systems, including cyber–physical systems, satellite systems, and IoT-aided aerial vehicle systems. By presenting a novel integration of blockchain technology into drone architecture, we address the concerns related to data security and unauthorized access that have plagued previous designs.

Finally, this research presents cutting-edge developments in drone technology and its intersection with the Internet of Things (IoT) and introduces a framework that significantly enhances security and resilience. Through a comparative analysis with prior works and an evaluation of our proposed approach on a merged dataset, we establish our study’s novel contributions and advancements, paving the way for more secure and efficient drone deployment in various fields.

## 6. Conclusions

The current study focused on proposing an IoT drone-based cyber-security framework network. This framework employs a voting ensemble of ML algorithms and employs data from various sources, such as network information, drones, and sensors, to identify security-level patterns and detect security attacks. The proposed architecture combines several cutting-edge technologies, such as machine learning, artificial intelligence, data fusion, and anomaly detection, to build a powerful and adaptable security solution. The framework can identify both known and unknown threats by utilizing the strength of advanced algorithms, allowing for quick response and mitigation actions.

The proposed framework was tried on the drone dataset and was able to demonstrate robust results for cyber-attack identification in real time, achieving an accuracy rate of 99.89%, which surpasses previous approaches. The performance of the proposed framework was evaluated on a newly constructed merged dataset in terms of accuracy, recall, precision, and F1-score. The RegressionNet model is proposed to accurately identify attack types and shows its authority and strength. This framework can be deployed to detect vulnerabilities in other domains as well in the future. Furthermore, in future work, we will also focus on adding a malware attack prevention layer in the proposed framework.

## Figures and Tables

**Table 1:** Frequently occurring data privacy and cyber-security threats to smart drones.

Attack	Cyber-Security Threats	Threats Found in	Countermeasures Introduced in
Protocol-based attacks	Security of communication link	[13,17,18,19]	[17]
	Data confidentiality protection	[9]	
	Replay attack	[20,21]	[22]
	Privacy leakage	[9,19]	
	De-authentication attack	[4,13]	
Sensor-based attacks	GPS-spoofing/-jamming attack	[12]	[23]
	Motion sensor spoofing	[24]	[25]
	UAV-spoofing/-jamming attack	[12]	
Compromised component	IoT security threats	[12]	
	Control/data interception	[12,18]	
Jammers	Denial of service	[4,12,13]	
	Stop packet delivery	[16]	[16]

**Table 2:** Machine learning for frequently occurring data privacy and cyber-security threats to smart drones.

Attack	Security Technique	Machine Learning Solution
Jamming	Secure offloading	Q-learning [42,44], DQN [47]
Denial of service	Secure offloading	Neural networks [41], Multivariate correlation analysis [48], Q-learning [49]
Malware	Access control	Q/Dyna-Q/PDS [50], K-nearest neighbors [51], Random Forest [51]
Intrusion	Access control	Naive Bayes [43], Support vector machine [43], neural network [52], K-NN [53]
Spoofing	Authentication	SVM [54], DNN [55], Dyna-Q [56], Q-learning [56]
Traffic blockage	Authentication	Q-learning [57]

**Table 3:** Details of classes in the dataset.

Class	Description
DoS Attack	Use of resources or services is denied to authorized users.
Normal	Connections are generated by simulating user behavior.
User-to-remote attacks	Access to account types of administrator is gained by unauthorized entities.
Prob attack	Information about the system is exposed to unauthorized entities.
Remote-to-local attacks	Access to hosts is gained by unauthorized entities.

**Table 4:** Description of machine learning models.

Model	Description
RF	RF is a classification algorithm employing Decision Trees or estimators in ensemble learning. It utilizes the bagging technique and bootstrap samples to train the trees. The results of the individual trees are combined by voting to improve the overall accuracy. All trees are constructed based on the same pattern to test the data to evaluate the model’s performance. A Decision Tree with a lower error rate is assigned a higher weight, which reduces the likelihood of a false prediction [66].
DT	DT is an ML model that is widely used for the classification of text, and it relies on multiple variables to make predictions about an independent capricious event. Data are fragmented into branches in it to construct a reversed tree, which consists of internal nodes, root nodes, and leaf nodes. This algorithm can efficiently handle every type of dataset without requiring a complex parametric structure [67].
NB	The NB classifier is a Bayes theorem-based classifier that assumes objectivity between conjecturers. This theorem serves as the foundation for the classifier and is easy to construct, requiring only simple iterative parameter estimation. As a result, it is well suited to large datasets. Despite its simplicity, the Naive Bayes classifier produces excellent results and outperforms other classifiers of sophisticated nature [68].
SVM	SVM is a popular algorithm for text classification that draws hyperplanes by maximizing the marginal distance to separate classes [69]. In binary classification, the text is divided into two non-overlapping classes by the SVM hyperplane. Compared with deep learning methods, SVM is simpler and less complex, making it easy to interpret. In addition to text classification, intrusion detection is also performed using SVM [70].

**Table 5:** Hyperparameter settings for ML models.

Classifier	Parameter
ET	Number of trees = 200, random state = 52, maximum depth = 15
DT	Number of trees = 200, random state = 52, maximum depth = 15
RF	Number of trees = 200, random state = 52, maximum depth = 15
LR	Solver = ‘lbfgs’, penalty = ‘l2’
SVM	C = 1.0, kernel = ‘rbf’, gamma = ‘auto’
NB	Binarize = 0.0, alpha = 1.0
MLP	Hidden layers = 3, neurons = 200, activation function = ‘reLU’, batch size = 16, dropout rate = 0.5, optimizer = ‘adam’
VC (LR + MLP)	Voting = ‘soft’

**Table 6:** Performance evaluation parameters.

Evaluation Parameter	Formula
Accuracy	$\frac{TP+TN}{TP+TN+FP+FN}$
Precision	$\frac{TP}{TP+FP}$
Recall	$\frac{TP}{TP+FN}$
F1-score	2*$\frac{precision.recall}{precision+recall}$

**Table 7:** Experimental setup for the proposed system.

Element	Details
Language	Python 3.8
OS	64-bit window 10
RAM	32 GB
GPU	Nvidia, 1060, 8 GB
CPU	Intel Xeon eight-core CPUs with 2.8 GHz processor

**Table 8:** Comparison of the proposed approach and other learning models.

Model	Accuracy	Precision	Recall	F1-Score
Random Forest	99.15%	99.82%	99.86%	99.84%
Decision Tree	99.11%	99.11%	99.21%	99.16%
Logistic Regression	99.53%	99.82%	99.90%	99.86%
Naive Bayes	97.32%	98.41%	97.27%	97.89%
Support Vector Machine	99.14%	99.22%	99.30%	99.26%
MLP	99.64%	99.76%	99.88%	99.82%
RegressionNet	99.80%	99.81%	99.89%	99.86%

**Table 9:** Detail of STIN dataset.

Domain	Attack Type	Attack Time
Terrestrial attacks	Web attack	15:21→15:31
	Botnet	15:01→15:10
	LDAP DDoS	16:01→16:11
	Backdoor	15:41→15:52
	NetBIO DDoS	16:41→16:50
	MSSQL DDoS	16:21→16:30
	Portmap DDoS	17:01→17:13
	UDP DDoS	17:41→17:52
	Syn DDoS	17:21→17:32
Satellite attacks	DUP DDoS	16:52→17:20
	Syn DDoS	15:23→15:57

**Table 10:** Accuracy of classifiers on STIN security and the merger of all datasets.

Attack Type	Accuracy
**Terrestrial Attack**	
UDP_Dos	100.00%
Syn_DDoS	95.81%
Average accuracy	97.90%
**Satellite Attack**	
Backdoor	97.41%
LDAP DDoS	94.22%
MSSQL DDoS	96.24%
NetBIO DDoS	97.37%
Portmap DDoS	92.19%
Syn DDoS	98.41%
UDP DDoS	98.99%
Average accuracy	97.90%
**Merged Dataset**	
All attacks	91.64%

**Table 11:** Performance comparison of the proposed approach and state-of-the-art models.

Method	Dataset	Accuracy
RegressionNet	Drone dataset	99.89%
RegressionNet	KDD CUP 99	99.87%
RegressionNet	NSL-KDD	99.90%
PCA + MCA [74]	KDD CUP 99	94.20%
Deep Neural Model [75]	KDD CUP 99	92.49%
DT-RFE [76]	KDD CUP 99	99.21%
SVM-ANN [77]	NSL-KDD	91.48%
Deep Hierarchical Model [78]	NSL-KDD	83.58%
DT-RFE [76]	NSL-KDD	99.23%

## Data Availability

The dataset utilized in this research is publicly available and can also be requested from the authors.
